# Role of Diagnostic Nerve Blocks in the Goal-Oriented Treatment of Spasticity with Botulinum Toxin Type A: A Case–Control Study

**DOI:** 10.3390/toxins16060258

**Published:** 2024-06-03

**Authors:** Mirko Filippetti, Stefano Tamburin, Rita Di Censo, Martina Adamo, Elisa Manera, Jessica Ingrà, Elisa Mantovani, Salvatore Facciorusso, Marco Battaglia, Alessio Baricich, Andrea Santamato, Nicola Smania, Alessandro Picelli

**Affiliations:** 1 Department of Neurosciences, Biomedicine and Movement Sciences, University of Verona, 37100 Verona, Italy; mirko.filippetti@univr.it (M.F.); rita.dicenso@univr.it (R.D.C.); martina.adamo22@gmail.com (M.A.); elisa.manera@gmail.com (E.M.); elisa.mantovani@univr.it (E.M.); nicola.smania@univr.it (N.S.); alessandro.picelli@univr.it (A.P.); 2 Spasticity and Movement Disorders ‘ReSTaRt’ Unit, Physical Medicine and Rehabilitation Section, Policlinico Riuniti Hospital, University of Foggia, 71122 Foggia, Italy; s.facciorusso89@gmail.com (S.F.); andrea.santamato@unifg.it (A.S.); 3 Physical and Rehabilitative Medicine, Department of Health Sciences, University of Eastern Piedmont “A. Avogadro”, 28100 Novara, Italy; marco.battaglia@uniupo.it (M.B.); alessio.baricich@med.uniupo.it (A.B.); 4 Canadian Advances in Neuro-Orthopedics for Spasticity Consortium (CANOSC), Kingston, ON K7K 1Z6, Canada

**Keywords:** spasticity, neurorehabilitation, diagnostic nerve block, botulinum neurotoxin-A, personalized treatment, goal-setting process

## Abstract

The goal-setting process is pivotal in managing patients with disabling spasticity. This case–control study assessed the role of diagnostic nerve blocks in guiding the goal-setting process within goal-targeted treatment of spasticity with botulinum neurotoxin-A. In this case–control study, patients with disabling spasticity underwent either a goal-setting process based on the patient’s needs and clinical evaluation (control group) or additional diagnostic nerve block procedures (case group). All enrolled patients underwent a focal treatment with botulinum neurotoxin-A injection and a 1-month follow-up evaluation during which goal achievement was quantified using the goal attainment scaling-light score system. Data showed a higher goal achievement rate in the case group (70%) than in the control group (40%). In conclusion, diagnostic nerve blocks may help guide the goal-setting process within goal-targeted treatment of spasticity with botulinum neurotoxin-A towards more realistic and achievable goals, thereby improving the outcomes of botulinum neurotoxin-A injection. Future studies should better explore the role of diagnostic nerve blocks to further personalize botulinum neurotoxin-A according to individual patients’ preferences and requirements.

## 1. Introduction

Post-stroke spasticity (PSS) is a frequent disabling complication that affects functions, activities, and community participation of patients. Spasticity is experienced by approximately 34% of stroke survivors within 18 months following a stroke, and peaks at 84% among patients with multiple sclerosis with a long disease history [1,2,3].

Botulinum neurotoxin-A (BoNT-A) injections have an A level of evidence for the treatment of upper and lower limb spasticity in patients with stroke and multiple sclerosis [4,5,6]. There are different techniques for guiding and assisting BoNT-A injection, and ultrasound is largely applied to this aim [7]. Diagnostic nerve blocks (DNBs) play many pivotal roles in managing patients with spasticity, and guidelines regarding their safety have been recently published [8]. DNBs may support the differential diagnosis between spastic hypertonia and other conditions when the clinical examination is not conclusive [8]. Furthermore, DNBs help predict outcomes of BoNT-A treatment for spasticity and better tailoring this treatment to the needs of individual patients [9,10]. There is a broad consensus on the use of DNBs in the assessment and treatment of some spasticity patterns (i.e., equinus varus foot) [11]. Better identification of how and when to treat spasticity may ameliorate outcomes, e.g., reduce pain, disability, and other consequences of spasticity [12]. The treatment should be goal-targeted following the patient’s issues and clinical manifestations. Treatment should address passive (e.g., improving positioning and orthotic compliance and reducing spasms and pain) and active (e.g., improving upper and lower limb function and activities of daily living and reducing disability and dependence) limitations according to the level of functioning of individual patients [12,13]. Treatment of spasticity more frequently targets activity and participation [14], although achieving active goals may be more challenging [15]. The decision-making process in the treatment of spasticity with BoNT-A should be tailored to individual patients and address specific, measurable, attainable, realistic, and timed (SMART) goals [16]. Factors predicting the outcomes of spasticity treatment with BoNT-A based on SMART goals have been seldom studied.

The present study aims to assess the possible role of DNBs in guiding the goal-setting process within goal-targeted treatment of spasticity with BoNT-A according to the SMART framework and the specific needs of individual patients.

## 2. Results

This case–control study included 40 patients with disabling spasticity. In the case group, we prospectively enrolled 20 consecutive patients (post-stroke: n = 12, multiple sclerosis: n = 8). The retrospective control group included 20 patients (post-stroke: n = 16, multiple sclerosis: n = 4). The two groups did not differ for the main clinical and demographic variables (Table 1). The mean total BoNT-A dose was slightly lower in the case group than it was in the control group, while the mean upper and lower limb BoNT-A was slightly higher in the case group, because the number of patients who had their upper and lower limb muscles injected differed between groups. Within the case group, 4 patients received BoNT-A treatment in the upper limb, 12 in the lower limb, and 4 in both sites. Within the control group, 2 patients received BoNT-A treatment in the upper limb, 5 in the lower limb, and 13 in both sites.

Passive goals were chosen by 13 and 10 patients in the case and control groups, respectively, whereas 7 and 10 patients in the case and control groups, respectively, chose active goals. The single goals chosen by patients in each group are detailed in Table 2.

All patients in both groups demonstrated at baseline some function on the goal chosen. The GAS-light score was significantly different between groups (control group: −0.70 ± 1.30; median, −1, interquartile range (IQR) [–2, 0]; case group: 0.25 ± 1.29, median, 0 IQR [–1, 1]; *p* = 0.026) with an effect size of 0.733. The achievement rate was significantly higher in the case group than it was in the control group (case: 70%; control: 40%; *p* = 0.028; Table 3). Of 20 patients in the case group, 9 achieved a better outcome than expected (i.e., a GAS-light score of +1 or +2) in comparison to 3 of 20 patients in the control group (Table 3). The rate of goal achievement in the passive domain (impairment/symptoms) was 69.2% (i.e., 9/13) in the case group and 50.0% (i.e., 5/10) in the control group. The rate of goal achievement in the active domain (activities/function) was 71.4% (i.e., 5/7) in the case group and 30.0% (i.e., 3/10) in the control group.

## 3. Discussion

The DNB is a safe procedure that can be performed in the neurorehabilitation medicine setting and plays an increasingly key role in the management of patients with spasticity. Our data add to this notion, in that they document that DNBs may not only predict the effect of BoNT-A treatment on injected muscles in patients with disabling spasticity, but may also allow better achievement of treatment goals according to the requirements and preferences of individual patients according to the SMART framework [16,18].

In more detail, we found that prespecified goals were reached in nearly twice as many patients with spasticity when the treating physician choose the BoNT-A injection strategy based on DNBs and clinical assessments (i.e., a 70% achievement rate) rather than clinical assessments only (i.e., a 40% achievement rate). This difference was significant, with a moderate-to-high effect. The main reason for this significant difference can be explained by the role of the DNB, which allows a transient functional clinical evaluation of the effects of BoNT-A injection prior to treatment [8,10], in particular, in more complex patients [9]. Indeed, we may hypothesize that choosing passive and active goals combining patients’ preferences and requirements, clinical evaluation, and DNBs may allow prioritizing goals based on a better a priori definition of their achievability.

Active goals in the activities/function domain have been reported to be more challenging to achieve than passive ones in the impairment/symptom domain, probably because they reflect patients’ desire to prioritize a desirable goal rather than an achievable one [15]. Our data indicate that active goals were chosen less frequently than passive ones in the control group, but DNBs helped achieve them more frequently.

Our data indicate that other potentially biasing factors did not influence the outcomes of this study, in that demographic, clinical, and BoNT-A injection baseline features were comparable between the two groups.

This study was open to some consideration and suffered from some limitations. First, the control group was retrospective, because all patients with disabling spasticity currently undergo DNB procedures in our clinical practice. Second, we included patients with spasticity after stroke and due to multiple sclerosis, who may have different requirements. Third, active goals within the activities/function domain were mostly chosen by patients with lower limb spasticity, impeding the generalization of results to upper limb function. Fourth, performing a DNB requires a long learning curve, and its use is not currently widespread, thus limiting its use to tertiary centers.

## 4. Conclusions

This study confirmed that applying clinical goals to spasticity treatment is an effective method to identify treatment priorities and personalize BoNT-A treatment [13,19,20,21]. The new findings of our study are that DNBs may further optimize the goal-setting process for BoNT-A treatment of patients with disabling spasticity towards more realistic and achievable goals. Future studies should explore the role of DNBs in these patients’ treatments, whether its therapeutic yield to BoNT-A injection is comparable across different disease stages, and if it may help in understanding if other treatments (e.g., intrathecal baclofen, phenol neurolysis, and spasticity-reducing surgery) [22] may be more appropriate, alone or in combination with BoNT-A, to achieve a specific goal. Multicenter randomized controlled trials may help answer these questions.

## 5. Materials and Methods

### 5.1. Study Design and Assessment Times

This case–control study enrolled consecutive patients (April 2023–January 2024) with disabling spasticity as a prospective group (cases) and compared them with a retrospective group (controls) set up from a chart review process (January 2020–December 2021). We used a retrospective control group because in our spasticity outpatient service DNBs are currently the standard procedure for muscle target identification for BoNT-A injection. Disabling spasticity was defined using clinical expertise and incorporating classification of functioning, disability and health domains as spasticity that is perceived by the individual or caregivers as hindering body function, activities, and/or participation [23].

Assuming 33% goal achievement in the control group and 60% in the case group, the power analysis (G*Power, alpha = 0.05, power = 0.90, two-tailed) indicated a sample size of 38, which was rounded to 40 patients (case group = 20, control group = 20).

**Figure 1:**
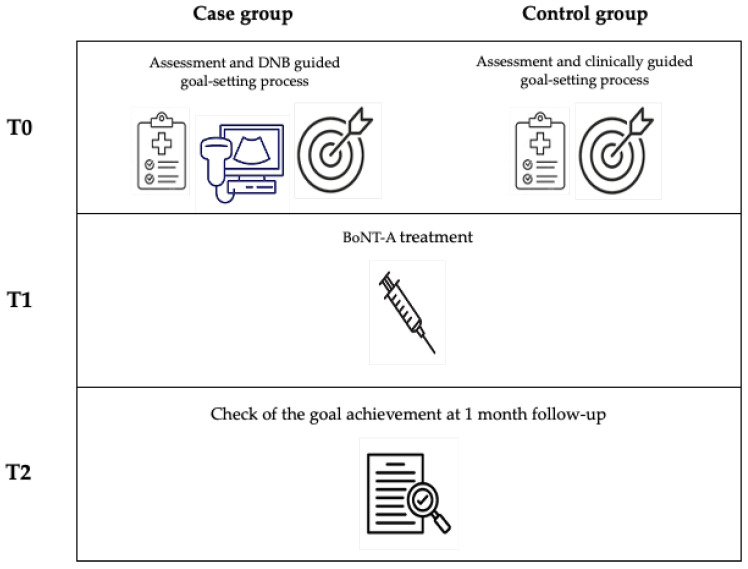
Flow chart of the study. DNB: diagnostic nerve block. BoNT-A: botulinum neurotoxin-A.

Inclusion criteria: (a) age > 18 years; (b) diagnosis of disabling spasticity of the upper and/or lower limb(s); (c) no contraindications to DNB (for the case group only) [8]; and (d) goal-setting process based on conventional clinical assessment (i.e., no DNB, for the control group only). Exclusion criteria: impossible to define a treatment goal and mini-mental state examination (MMSE) corrected for age and education < 23.8/30. The timing of assessment and treatment is described in Figure 1. At T0, patients in the case group underwent clinical evaluations and DNBs that guided the definition of their BoNT-A treatment goals. In contrast, only the clinical evaluation at T0 guided the definition of BoNT-A treatment goals for control patients. At T1, around 1–2 weeks later, all patients underwent treatment with BoNT-A injection, according to goals defined at T0. At T2, 1 month after T1, all patients underwent a follow-up visit to assess whether their goals were effectively achieved using the GAS-light scoring system. Patients did not undergo changes in oral therapy or other treatments for spasticity between T1 and T2. Figure 1 shows the flow-chart of this study.

### 5.2. Goal-Setting Process

**Figure 2:**
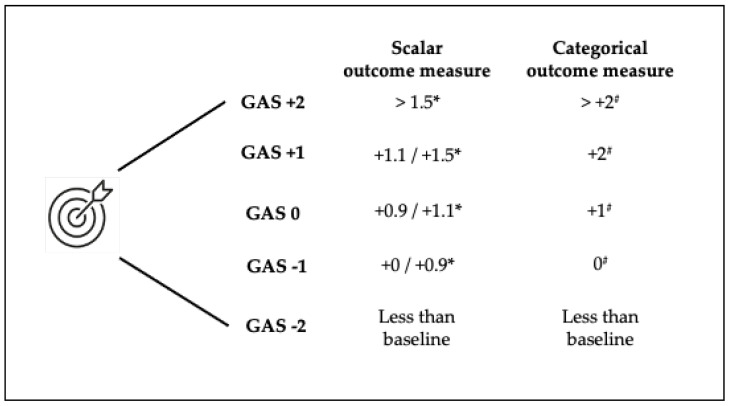
Scores of the goal attainment scale (GAS). * Gain expressed as number of times of minimal clinically important difference or minimal detectable change from the baseline. # Gain expressed in point(s) from the baseline.

The definition of each goal, referred to a function and matched to a specific outcome measure, was based on the requirements and preferences of each individual patient. A single goal was set for each individual patient. Possible goals, goal areas, and domains are listed in Table 4. Evaluation of the achievement of the chosen goal followed the GAS-light model [24] in accordance with the needs of each individual patient. The GAS-light model is based on six key steps (i.e., 1. What are the patient’s main problems? 2. What do you expect to be able to achieve? 3. Have the team and the patient/caregiver agreed on the expected outcome? 4. How will the outcome be assessed? 5. Plan treatment. 6. Review.), a baseline function evaluation of the goal (i.e., some function, no function), and a 5-point scoring system (i.e., goal achieved as expected, 0; a little more, +1; a lot more, +2; no change from baseline, −1; got worse, −2). The expected target was defined based on the SMART framework for each patient. GAS-light cut-off scores were based on the minimal clinical important difference (MCID) or minimal detectable change (MCD). For scalar measures, a GAS-light score of 0 was attributed if the gain from the baseline ranged between 0.9 and 1.1 times the MCD/MCID; a score of +1 was attributed if the gain ranged between 1.1 and 1.5 times the MCD/MCID; a score of +2 was attributed if the gain was more than 1.5 times the MCD/MCID; a score of −1 was attributed if no or minimal gain was achieved (i.e., less than 0.9 times the MCD/MCID); and a score of −2 was attributed if the outcome was worse than the baseline. For categorical outcome measures, score 0 was set as a gain from the baseline equal to 1 point; score −1 if there was no change from the baseline; score −2 if the outcome was worse than the baseline; score +1 when there was an improvement of at least 2 points; and score +2 when there was an improvement of more than 2 points. Goals, their respective outcome measures, and the GAS-light score 0 are described in Table 4 and Figure 2. Decimal values were rounded up if ≥ 0.5 and rounded down if < 0.5, except for seconds, where the sensitivity of the chronometer was set at 0.1” and rounded up/down if ≥/< 0.05.

### 5.3. DNB Procedures

DNBs were performed with a 22-gauge, 80-mm, ultrasound faceted tip echogenic needle for nerve block (SonoPlex STIM, Pajunk, Geisingen, Germany) applied to the motor branches of the target nerve by ultrasound (MyLab 70 XVision system, Esaote SpA, Genoa, Italy; linear probe set at 13 MHz) using an in-plane technique and electrical nerve stimulation (Plexygon, Vygon, Padua, Italy) guidance. Patients laid in a prone or supine position as appropriate. When the target nerve was identified by ultrasound, and following elicitation of appropriate muscle response to electrical stimulus (1 Hz, 100 μs, 0.5 mA), lidocaine (2%) was injected [33]. The maximum dose administered during each session of DNB was 2 mg/kg, following French clinical guidelines for peripheral motor nerve blocks in a physical and rehabilitation medicine setting [8]. DNB targets for the upper limbs included the lateral and medial pectoral nerves, the musculo-cutaneus nerve, and median and ulnar nerves. DNB lower limb targets included the anterior obturator nerve, femoral nerve and its motor branches for the rectus femoris muscle, and the tibialis nerve main trunk and its motor branches [9,34]. Each nerve was identified according to previously described techniques [35,36]. The amount of lidocaine changed according to the type of DNB, i.e., 1–2 mL for muscular motor branches (e.g., motor branch for the soleus muscle), 3–4 mL for main trunk nerves (e.g., the median nerve), and 5–6 mL for the tibial nerve. DNB procedures were performed by medical doctors with more than five years of experience in managing this technique.

### 5.4. BoNT-A Treatment

The BoNT-A injection strategy was chosen by the treating physician according to clinical and DNB evaluations (case group), or the clinical evaluation only (control group), and was verified one month after BoNT-A treatment at T2. The choice of injected muscles was based on the spasticity pattern of upper/lower limbs for each patient [37,38,39]. BoNT-A injections were guided by ultrasound (MyLab 70 XVision system, Esaote SpA, Genoa, Italy; linear probe set at 13 MHz) using a 22-gauge, 40 mm needle. Injection sites were identified according to the literature [40,41]. The dilution was 100 U/2 mL for onabotulinumtoxin-A and incobotulinumtoxin-A, and 500 U/2.5 mL for abobotulinumtoxin-A. Dosages, the number of injection sites, and total dose per session were in accordance with the product information for each BoNT-A type.

### 5.5. Statistical Analysis

Statistical analysis was performed with the SPSS version 21.0 (SPSS, Chicago, IL, USA) package. For continuous variables, normality of distribution was tested with the Shapiro–Wilks test. A descriptive analysis of collected data was performed using the mean, standard deviation, and median and interquartile ranges, as appropriate. Based on the study’s design, with two groups, but a single measure for both the baseline demographic, clinical, and treatment characteristics data (Table 1) and for the primary outcome (i.e., the GAS-light score), an independent samples t-test was used in cases of normal distribution, while the non-parametric Mann–Whitney U test was applied when the distribution was not normal (*p* < 0.05, two-tailed). Cohen’s d was used for the calculation of the effect size. The chi-squared (χ2) test was applied when comparing the rate of achievement between case and control groups (*p* < 0.05, one-tailed).

## Figures and Tables

**Table 1:** Demographic, clinical, and treatment characteristics of patients with disabling spasticity.

	Case Group (n = 20)	Control Group (n = 20)	t, χ2 Values	*p*
Demographic				
Age, years	58.0 ± 16.7	62.5 ± 12.3	t(38) = 0.97	0.34
Male, %	50.0	70.0	χ2 (1,1) = 1.67	0.20
Clinical			χ2 (1,1) = 1.07	0.30
Multiple sclerosis, %	40.0	20.0		
Post-stroke, %	60.0	80.0		
Time from stroke onset, mos	113.6 ± 108.1	64.5 ± 52.2	t(26) = 1.59	0.12
Time from spasticity diagnosis, mos	96.1 ± 60.7	70.5 ± 39.5	t(12) = 0.77	0.46
BoNT-A treatment				
Total BoNT-A dose, unit *	292.3 ± 109.9	320.4 ± 139.2	t(36) = −0.70	0.49
Upper limb BoNT-A dose, unit *	199.0 ± 84.71	152.9 ± 56.1	t(31) = 1.87	0.07
Lower limb BoNT-A dose, unit *	264.4 ± 94.6	211.9 ± 71.8	t(31) = 1.80	0.08
Injected muscles, N	3.5 ± 1.6	4.7 ± 2.4	t(36) = −1.93	0.06
Upper limb injected muscles, N	3.1 ± 1.4	2.8 ± 0.8	t(21) = 0.56	0.58
Lower limb injected muscles, N	2.8 ± 1.3	2.8 ± 1.3	t(31) = −0.02	0.98

Legend. BoNT-A: botulinum neurotoxin-A; mos = months; N = number; * onabotulinum toxin-A and incobotulinum toxin-A to abobotulinum toxin-A ratio: 1:3 [17].

**Table 2:** Goals chosen by patients in each group.

Domain	Goal Areas	Goals	Outcome Measures	Case Group	Control Group
Passive goals (impairment/ symptoms)	Pain/Discomfort	Pain reduction	NPRS	2	2
	Involuntary movements	Spasms reduction	PSFS	4	2
		Clonus reduction	Tardieu score	1	1
	Range of movement/ contractures prevention	Muscle tone reduction	MAS	4	2
		Gain in ankle pROM	angle	1	3
		Gain in shoulder abduction pROM	angle	1	0
Active goals (activities/ function)	Active function	Gain in muscle strength	MRC	0	0
		Gain in balance during gait	TUG	2	0
		Gain in hand dexterity	NHPT	0	0
	Mobility	Gain in gait endurance	6 MWT	1	3
		Gain in gait speed	10 MWT	2	3
		Gain in gait functioning	LegA	2	4

Legend. NPRS: numerical pain rating scale; PSFS: Penn spasm frequency scale; MAS: modified Ashworth scale; pROM: passive range of motion; MRC: medical research council scale; TUG: time up and go test; NHPT: nine-hole peg test; 6 MWT: 6 min walking test; 10 MWT: 10 m walking test; LegA: leg activity.

**Table 3:** GAS-light scoring system.

GAS-Light Scoring System		Case Group	Control Group	Rate of Achievement (Case Group)	Rate of Achievement (Control Group)
Some function		20	20	-	-
No function		0	0		
Not achieved	score −2	2	7	30%	60%
	score −1	4	5		
Achieved	score 0	5	5	70% *	40%
	score +1	5	1		
	score +2	4	2		

Legend. GAS: goal attainment scale. * Significantly higher than control group; χ2: *p* = 0.028.

**Table 4:** Goals, outcome measures, and expected targets.

Domain	Goal Areas	Goals	Outcome Measures	Expected Targets GAS 0
Passive goals (impairment/symptoms)	Pain/discomfort	Pain reduction	NPRS	−1 point [25]
	Involuntary movements	Spasms reduction	PSFS	−1 point *
		Clonus reduction	Tardieu score	−1 point [26]
	Range of movement/ contractures prevention	Muscle tone reduction	MAS	−1 point [27]
		Gain in ankle pROM	angle	+7.7° [28]
		Gain in shoulder abduction pROM	angle	+15° *#
Active goals (activities/function)	Active function	Gain in muscle strength	MRC	+1 point *
		Gain in balance during gait	TUG	−2.9′′ [29]
		Gain in hand dexterity	NHPT	−32.8′′ [30]
	Mobility	Gain in gait endurance	6 MWT	+34.4 m [31]
		Gain in gait speed	10 MWT	+0.16 m/s [32]
		Gain in gait functioning	LegA	−13.2 points *^$^

Legend. GAS: goal attainment scale; NPRS: numerical pain rating scale; PSFS: Penn spasm frequency scale; MAS: modified Ashworth scale; pROM: passive range of motion; MRC: medical research council scale; TUG: time up and go test; NHPT: nine-hole peg test; 6 MWT: 6 min walking test; 10 MWT: 10 m walking test; LegA: leg activity. * No minimal clinically important difference or minimal detectable change was available. ^$^ The target was set as an improvement from a baseline of 13 points, representing 10% of the maximum achievable score (i.e., 132 points). # The target was set as the difference from a baseline of 15°, which represents 10% of the complete range of movement (i.e., 150°).

## Data Availability

Original data are available by contacting the corresponding authors upon reasonable request.

## References

[1] Watkins C.L., Leathley M.J., Gregson J.M., Moore A.P., Smith T.L., Sharma A.K. (2002). Prevalence of spasticity post stroke. Clin. Rehabil..

[2] Bethoux F., Marrie R.A. (2016). A Cross-Sectional Study of the Impact of Spasticity on Daily Activities in Multiple Sclerosis. Patient.

[3] Rizzo M.A., Hadjimichael O.C., Preiningerova J., Vollmer T.L. (2004). Prevalence and treatment of spasticity reported by multiple sclerosis patients. Mult. Scler..

[4] Otero-Romero S., Sastre-Garriga J., Comi G., Hartung H.P., Soelberg Sørensen P., Thompson A.J., Vermersch P., Gold R., Montalban X. (2016). Pharmacological management of spasticity in multiple sclerosis: Systematic review and consensus paper. Mult. Scler..

[5] Dressler D., Bhidayasiri R., Bohlega S., Chahidi A., Chung T.M., Ebke M., Jacinto L.J., Kaji R., Koçer S., Kanovsky P. (2017). Botulinum toxin therapy for treatment of spasticity in multiple sclerosis: Review and recommendations of the IAB-Interdisciplinary Working Group for Movement Disorders task force. J. Neurol..

[6] Simpson D.M., Hallett M., Ashman E.J., Comella C.L., Green M.W., Gronseth G.S., Armstrong M.J., Gloss D., Potrebic S., Jankovic J. (2016). Practice guideline update summary: Botulinum neurotoxin for the treatment of blepharospasm, cervical dystonia, adult spasticity, and headache: Report of the Guideline Development Subcommittee of the American Academy of Neurology. Neurology.

[7] Asimakidou E., Sidiropoulos C. (2023). A Bayesian Network Meta-Analysis and Systematic Review of Guidance Techniques in Botulinum Toxin Injections and Their Hierarchy in the Treatment of Limb Spasticity. Toxins.

[8] Yelnik A.P., Hentzen C., Cuvillon P., Allart E., Bonan I.V., Boyer F.C., Coroian F., Genet F., Honore T., Jousse M. (2019). French clinical guidelines for peripheral motor nerve blocks in a PRM setting. Ann. Phys. Rehabil. Med..

[9] Winston P., Reebye R., Picelli A., David R., Boissonnault E. (2023). Recommendations for Ultrasound Guidance for Diagnostic Nerve Blocks for Spasticity. What Are the Benefits?. Arch. Phys. Med. Rehabil..

[10] Deltombe T., Wautier D., De Cloedt P., Fostier M., Gustin T. (2017). Assessment and treatment of spastic equinovarus foot after stroke: Guidance from the Mont-Godinne interdisciplinary group. J. Rehabil. Med..

[11] Salga M., Gatin L., Deltombe T., Gustin T., Carda S., Marque P., Winston P., Reebye R., Wein T., Esquenazi A. (2023). International Recommendations to Manage Poststroke Equinovarus Foot Deformity Validated by a Panel of Experts Using Delphi. Arch. Phys. Med. Rehabil..

[12] Francisco G.E., McGuire J.R. (2012). Poststroke spasticity management. Stroke.

[13] Newsome S.D., Thrower B., Hendin B., Danese S., Patterson J., Chinnapongse R. (2022). Symptom burden, management and treatment goals of people with MS spasticity: Results from SEEN-MSS, a large-scale, self-reported survey. Mult. Scler. Relat. Disord..

[14] Choi K., Peters J., Tri A., Chapman E., Sasaki A., Ismail F., Boulias C., Reid S., Phadke C.P. (2017). Goals Set by Patients Using the *ICF* Model before Receiving Botulinum Injections and Their Relation to Spasticity Distribution. Physiother. Can..

[15] Singh R., Clarke A. (2020). Real-life outcomes in spasticity management: Features affecting goal achievement. BMJ Neurol. Open.

[16] Picelli A., Battistuzzi E., Filippetti M., Modenese A., Gandolfi M., Munari D., Smania N. (2020). Diagnostic nerve block in prediction of outcome of botulinum toxin treatment for spastic equinovarus foot after stroke: A retrospective observational study. J. Rehabil. Med..

[17] Scaglione F. (2016). Conversion Ratio between Botox^®^, Dysport^®^, and Xeomin^®^ in Clinical Practice. Toxins.

[18] Jacinto J., Balbert A., Bensmail D., Carda S., Draulans N., Deltombe T., Ketchum N., Molteni F., Reebye R. (2023). Selecting Goals and Target Muscles for Botulinum Toxin A Injection Using the Goal Oriented Facilitated Approach to Spasticity Treatment (GO-FAST) Tool. Toxins.

[19] Turner-Stokes L., Jacinto J., Fheodoroff K., Brashear A., Maisonobe P., Lysandropoulos A., Ashford S., Upper Limb International Spasticity-III (ULIS-III) study group (2021). Assessing the effectiveness of upper-limb spasticity management using a structured approach to goal-setting and outcome measurement: First cycle results from the ULIS-III Study. J. Rehabil. Med..

[20] Ashford S., Williams H., Nair A., Orridge S., Turner-Stokes L. (2019). Categorisation of goals set using Goal Attainment Scaling for treatment of leg spasticity: A multicentre analysis. Disabil. Rehabil..

[21] Baccouche I., Bensmail D., Leblong E., Fraudet B., Aymard C., Quintaine V., Pottier S., Lansaman T., Malot C., Gallien P. (2022). Goal-Setting in Multiple Sclerosis-Related Spasticity Treated with Botulinum Toxin: The GASEPTOX Study. Toxins.

[22] Tamburin S., Filippetti M., Mantovani E., Smania N., Picelli A. (2022). Spasticity following brain and spinal cord injury: Assessment and treatment. Curr. Opin. Neurol..

[23] Biering-Soerensen B., Stevenson V., Bensmail D., Grabljevec K., Martínez Moreno M., Pucks-Faes E., Wissel J., Zampolini M. (2022). European expert consensus on improving patient selection for the management of disabling spasticity with intrathecal baclofen and/or botulinum toxin type A. J. Rehabil. Med..

[24] Clarkson K., Barnett N. (2021). Goal attainment scaling to facilitate person-centred, medicines-related consultations. Eur. J. Hosp. Pharm..

[25] Salaffi F., Stancati A., Silvestri C.A., Ciapetti A., Grassi W. (2004). Minimal clinically important changes in chronic musculoskeletal pain intensity measured on a numerical rating scale. Eur. J. Pain.

[26] Paulis W.D., Horemans H.L., Brouwer B.S., Stam H.J. (2011). Excellent test-retest and inter-rater reliability for Tardieu Scale measurements with inertial sensors in elbow flexors of stroke patients. Gait Posture.

[27] Shaw L., Rodgers H., Price C., van Wijck F., Shackley P., Steen N., Barnes M., Ford G., Graham L., BoTULS investigators (2010). BoTULS: A multicentre randomised controlled trial to evaluate the clinical effectiveness and cost-effectiveness of treating upper limb spasticity due to stroke with botulinum toxin type A. Health Technol. Assess..

[28] Konor M.M., Morton S., Eckerson J.M., Grindstaff T.L. (2012). Reliability of three measures of ankle dorsiflexion range of motion. Int. J. Sports Phys. Ther..

[29] Flansbjer U.B., Holmbäck A.M., Downham D., Patten C., Lexell J. (2005). Reliability of gait performance tests in men and women with hemiparesis after stroke. J. Rehabil. Med..

[30] Chen H.M., Chen C.C., Hsueh I.P., Huang S.L., Hsieh C.L. (2009). Test-retest reproducibility and smallest real difference of 5 hand function tests in patients with stroke. Neurorehabil. Neural Repair.

[31] Wise R.A., Brown C.D. (2005). Minimal clinically important differences in the six-minute walk test and the incremental shuttle walking test. COPD J. Chronic Obstr. Pulm. Dis..

[32] Tilson J.K., Sullivan K.J., Cen S.Y., Rose D.K., Koradia C.H., Azen S.P., Duncan P.W., Locomotor Experience Applied Post Stroke (LEAPS) Investigative Team (2010). Meaningful gait speed improvement during the first 60 days poststroke: Minimal clinically important difference. Phys. Ther..

[33] Filippetti M., Di Censo R., Varalta V., Baricich A., Santamato A., Smania N., Picelli A. (2022). Is the Outcome of Diagnostic Nerve Block Related to Spastic Muscle Echo Intensity? A Retrospective Observational Study on Patients with Spastic Equinovarus Foot. J. Rehabil. Med..

[34] Facciorusso S., Spina S., Gasperini G., Picelli A., Filippetti M., Molteni F., Santamato A. (2023). Anatomical landmarks for ultrasound-guided rectus femoris diagnostic nerve block in post-stroke spasticity. Australas. J. Ultrasound Med..

[35] Wu C.H., Chang K.V., Özçakar L., Hsiao M.Y., Hung C.Y., Shyu S.G., Wang T.G., Chen W.S. (2015). Sonographic tracking of the upper limb peripheral nerves: A pictorial essay and video demonstration. Am. J. Phys. Med. Rehabil..

[36] Hung C.Y., Hsiao M.Y., Özçakar L., Chang K.V., Wu C.H., Wang T.G., Chen W.S. (2016). Sonographic Tracking of the Lower Limb Peripheral Nerves: A Pictorial Essay and Video Demonstration. Am. J. Phys. Med. Rehabil..

[37] Simpson D.M., Patel A.T., Alfaro A., Ayyoub Z., Charles D., Dashtipour K., Esquenazi A., Graham G.D., McGuire J.R., Odderson I. (2017). OnabotulinumtoxinA Injection for Poststroke Upper-Limb Spasticity: Guidance for Early Injectors from a Delphi Panel Process. Pm R.

[38] Hefter H., Jost W.H., Reissig A., Zakine B., Bakheit A.M., Wissel J. (2012). Classification of posture in poststroke upper limb spasticity: A potential decision tool for botulinum toxin A treatment?. Int. J. Rehabil. Res..

[39] Esquenazi A., Alfaro A., Ayyoub Z., Charles D., Dashtipour K., Graham G.D., McGuire J.R., Odderson I.R., Patel A.T., Simpson D.M. (2017). OnabotulinumtoxinA for Lower Limb Spasticity: Guidance from a Delphi Panel Approach. PM R.

[40] Kara M., Kaymak B., Ulaşli A.M., Tok F., Öztürk G.T., Chang K.V., Hsiao M.Y., Hung C.Y., Yağiz On A., Özçakar L. (2018). Sonographic guide for botulinum toxin injections of the upper limb: EUROMUSCULUS/USPRM spasticity approach. Eur. J. Phys. Rehabil. Med..

[41] Kaymak B., Kara M., Tok F., Ulaşli A.M., Öztürk G.T., Chang K.V., Hsiao M.Y., Hung C.Y., Yağiz On A., Özçakar L. (2018). Sonographic guide for botulinum toxin injections of the lower limb: EUROMUSCULUS/USPRM spasticity approach. Eur. J. Phys. Rehabil. Med..

